# Therapy of corneal endothelial dysfunction with corneal endothelial cell-like cells derived from skin-derived precursors

**DOI:** 10.1038/s41598-017-13787-1

**Published:** 2017-10-17

**Authors:** Lin Shen, Peng Sun, Canwei Zhang, Le Yang, Liqun Du, Xinyi Wu

**Affiliations:** 1 Department of Ophthalmology, Qilu Hospital of Shandong University, Jinan, Shandong 250012 PR China; 2 The Key Laboratory of Cardiovascular Remodeling and Function Research, Chinese Ministry of Education and Chinese Ministry of Health, Qilu Hospital of Shandong University, Jinan, Shandong 250012 PR China; 3 Department of Vascular Surgery, Shandong Provincial Hospital Affiliated to Shandong University, Jinan, Shandong 250021 PR China

## Abstract

Corneal endothelial dysfunction occurs when corneal endothelial cells (CECs) are dramatically lost and eventually results in vision loss. Corneal transplantation is the only solution at present. However, corneal transplantation requires a fresh human cornea and there is a worldwide shortage of donors. Therefore, finding new functional CECs to replace human CECs is urgent. Skin-derived precursors (SKPs) can be easily acquired and have multiple differential potential. We co-cultured human SKPs with B4G12 cells in serum-free medium and obtained abundant CEC-like cells which had similar morphology and characteristic to human CECs. CEC-like cells exerted excellent therapeutic effect when they were transplanted into rabbit and monkey corneal endothelial dysfunction models by injection method. This protocol enables efficient production of CEC-like cells from SKPs. The renewable cell source, novel derivation method and simple treatment strategy may lead to potential applications in cell replacement therapy for corneal endothelial dysfunction.

## Introduction

Corneal endothelial cells (CECs) are a monolayer of hexagonal cells covering the posterior surface of the cornea, and serve as a barrier between the corneal stroma and the aqueous humor. The tight junction as well as the ionic “pump” functions of CECs are important factors that maintain corneal transparency^1^. Many pathological factors such as trauma, surgery, and inflammation may cause dramatic loss of endothelial cell density, resulting in corneal endothelial dysfunction which is characterized by corneal edema, bullous keratopathy, and loss of visual acuity^2^. Human CECs have limited proliferative capacity *in vivo* and cannot be subcultured for more than a few passages *in vitro* ^3,4^. Corneal transplantation is the only solution at present. However, corneal transplantation requires a fresh human cornea and there is a worldwide shortage of donors^5^. Therefore, finding new functional CECs to replace human CECs is a new and potential direction for future clinical application.

Skin-derived precursors (SKPs) were discovered by Tomas^6,7^, and they have been a research hotspot in recent years. SKPs can be isolated in large quantities from many parts of the skin in rodents and humans. As they are multipotent, they can be differentiated into several functional cell types^8^, and are able to suppress the allogeneic activation of T-lymphocytes resulting in an improved health status of animals suffering from a graft-versus-host reaction^9^. Moreover, SKPs were demonstrated to be embryonic neural-crest-related precursors and share many properties with neural crest stem cells^10^. CECs mainly originate from neural crest cells^11–13^, so this tissue similarity, together with easy accessibility and low immunogenicity make SKPs ideal seed cells be differentiated to CECs by mimicking the developmental process.

In the last decade, Descemet’s stripping endothelial keratoplasty (DSEK) and Descemet’s membrane endothelial keratoplasty (DMEK) have been used to treat corneal endothelial dysfunction^14^. But the surgical procedure is complex and difficult and the immunological rejection often occurs. In recent years, research reported that injecting cells into anterior chamber may be an alternative method and this method nearly does not cause adverse effects^15,16^.

In this study, we explored a feasible protocol of differentiating SKPs into CEC-like cells. We also injected CEC-like cells into rabbit and monkey corneal endothelial dysfunction models to test the CEC-like cell function *in vivo*. Results showed that CEC-like cells could be derived from SKPs, and they had similar morphology and characteristic to human CECs. Most importantly, our animal transplantation experiment was also very successful that the corneal endothelial dysfunction models recovered in a very short time. Especially in the monkey, the corneas remained in good condition after three months and there was no apparent immune rejection. Previously, Inagaki *et al*.^17^ had reported corneal endothelial-like cells could be derived from SKPs. Compared to their work, we use different derivation method, operation method and animal model. Our therapeutic schedule may be a clinically relevant cell-based therapy for treating corneal endothelial dysfunction in the future.

## Results

### The derivation of CEC-like cells from SKPs

**Figure 1:**
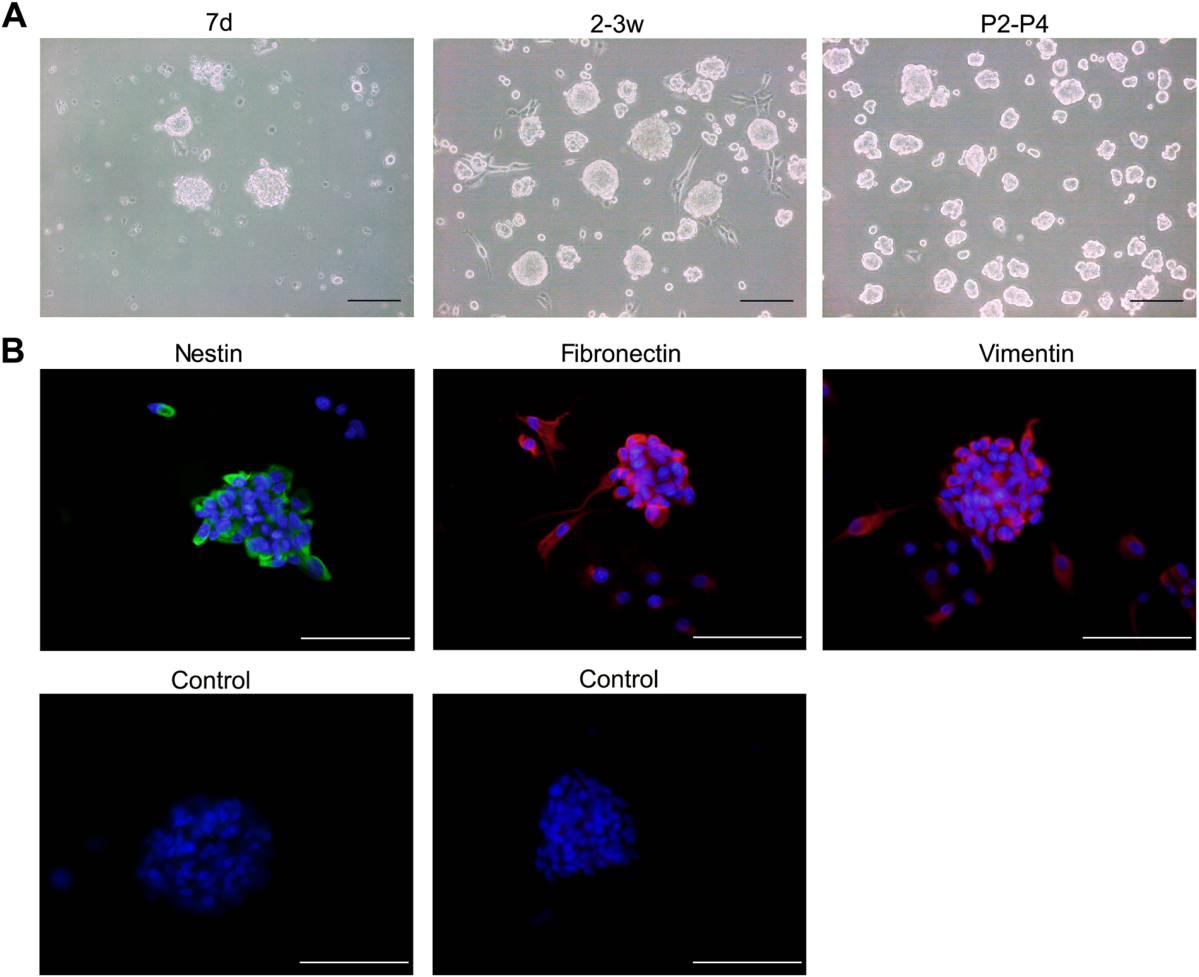
Cultivation of skin-derived precursors (SKPs). (**A**) SKPs were cultured and subcultured in the form of floating spheres. (**B**) SKPs expressed nestin, fibronectin, and vimentin. Scale bars 100 μm.

Human SKPs formed small floating spheres at day 3–7. Over 2–3 weeks a population of larger proliferating spheres formed. Purified populations of floating spheres were obtained using the process of selective adhesion. SKPs spheres were split into small spheres to proliferate when passaged every 1–2 weeks (Fig. 1A). Fluorescent immunochemical staining revealed that the spheres expressed nestin, fibronectin, and vimentin, which confirmed their identity as SKPs (Fig. 1B).

**Figure 2:**
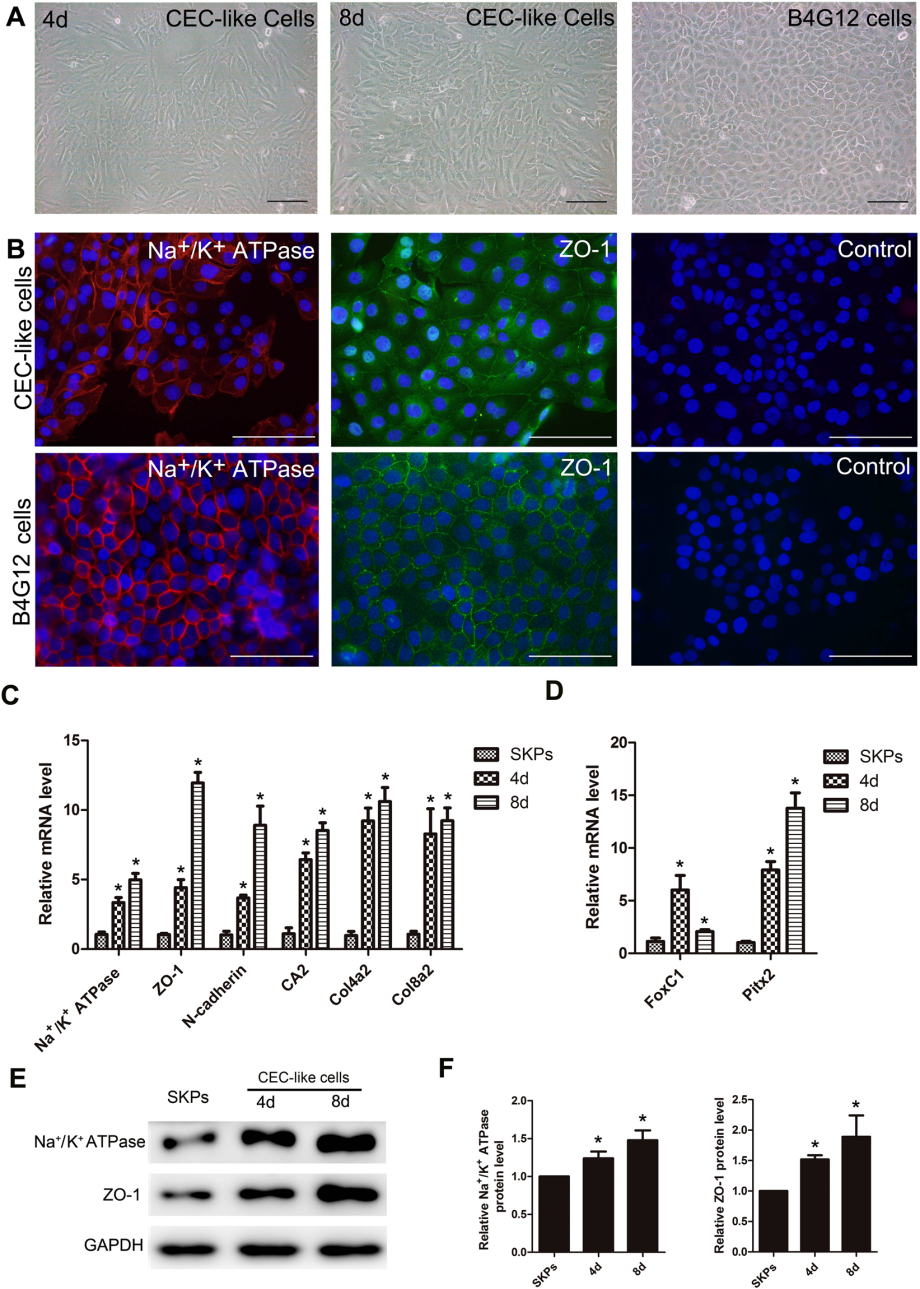
Derivation of CEC-like cells from SKPs. (**A**) During co-culturing, cell morphology began to change to a polygonal CEC-like shape on day 4. After 8 days, the polygonal CEC-like cells were the majority and formed a mosaic monolayer. (**B**) Immunofluorescent staining showed that the CEC-like cells expressed corneal endothelium major markers Na^+^/K^+^ ATPase and ZO-1. (**C**) RT-PCR analysis of corneal endothelial markers in CEC-like cells and SKPs. (**D**) Expression of Pitx2 and FoxC1 during derivation. (**E**,**F**) Western blotting analysis and protein levels of Na^+^/K^+^ ATPase alpha 1 and ZO-1 in CEC-like cells compared to SKPs. All samples derived from the same experiment were processed in parallel. One representative experiment out of three is presented. Cropped images were displayed and full-length blots are shown in the Supplementary Figure 4. Results are mean ± S.E.M. *p < 0.05 compared to SKPs.

The SKP spheres were dissociated by trypsin to single cells to be induced to CEC-like cells. After dissociation, SKPs adhered to the flask and had a spindle shape. During co-culturing with the B4G12 cells, the cell morphology began to change to a polygonal CEC-like shape on day 4. After 8 days, the polygonal CEC-like cells were the majority and formed a mosaic monolayer. We prolonged the induction time to 12 days, but no more increase of CEC-like cells was observed (Fig. 2A). Immunofluorescent staining showed that the CEC-like cells expressed corneal endothelium major markers Na^+^/K^+^ ATPase which acts as a pump and tight junction protein ZO-1(Fig. 2B). RT-PCR was carried out to detect the change of expression level of more markers including Na^+^/K^+^ ATPase alpha 1 (Atp1a1), tight junction protein 1 (ZO-1), N-cadherin, carbonic anhydrase type 2 (Car2), collagen type IV alpha 2 (Col4a2), and collagen type VIII alpha 2 (Col8a2). RT-PCR showed that CEC-like cells express significantly higher levels of all corneal endothelial markers and changed over time after derivation compared to SKPs (Fig. 2C). Paired-like homeodomain 2 (Pitx2) and forkhead box C1 (FoxC1) which are the transcription factors associated with human corneal endothelial development, also changed during derivation. Expression of FoxC1 peaked on day 4 and then quickly declined. Unlike FoxC1, expression of Pitx2 continued to rise during the period of observation (Fig. 2D). Western blot analysis also showed that CEC-like cells express higher protein levels of Na^+^/K^+^ ATPase alpha 1 and ZO-1 compared to SKPs (Fig. 2E,F).

**Figure 3:**
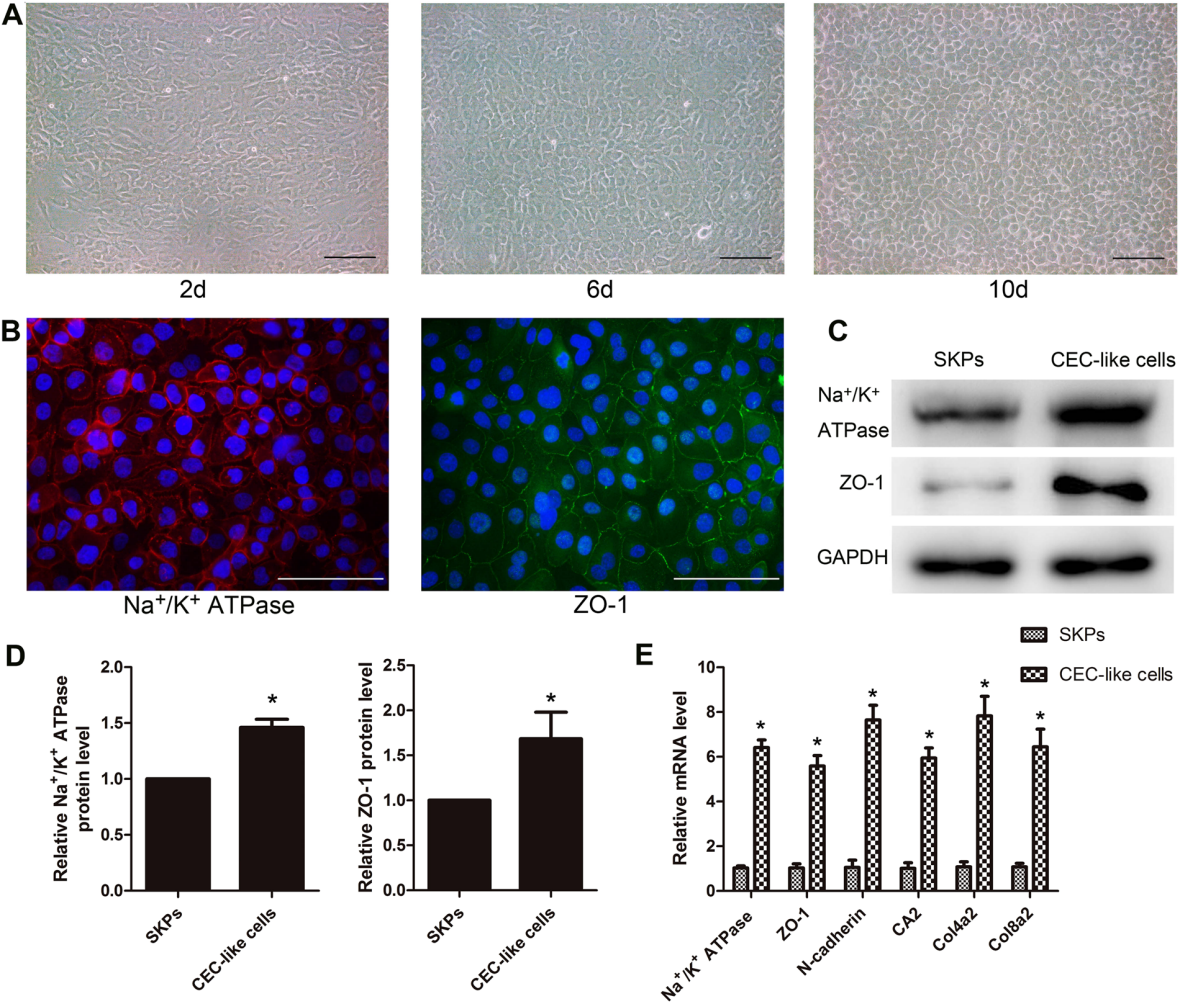
Characteristic of subcultured CEC-like cells. (**A**) Subcultured CEC-like cells formed a monolayer of hexagonal and pentagonal cells through 10 days of culture. (**B**–**E**) Immunofluorescent staining, Western blotting, and RT-PCR showed that the passaged CEC-like cells continued to express CECs markers. All samples derived from the same experiment were processed in parallel. One representative experiment out of three is presented. Cropped images were displayed and full-length blots are shown in the Supplementary Figure 5. Scale bars 100 μm. Results are mean ± S.E.M. *p < 0.05 compared to SKPs.

After co-culturing, CEC-like cells were passaged and cultured by human endothelial SFM supplemented with 10ng/ml bFGF. CEC-like cells could grow well, and had strong proliferation ability. They formed a monolayer of hexagonal and pentagonal cells through 10 days of culture (Fig. 3A). When the culturing time was extended to 1 month, the cell density and shape did not change, indicating the phenomenon of cell-to-cell contact inhibition. These biological features of CEC-like cells were similar to normal human CECs. Immunofluorescent staining, Western blotting, and RT-PCR showed that the passaged CEC-like cells continued to express Na^+^/K^+^ ATPase, ZO-1, and other CECs markers, indicating that the characteristics were maintained under current culture conditions (Fig. 3B–E).

### Clinical observations after transplantation of CEC-like cells in rabbits

**Figure 4:**
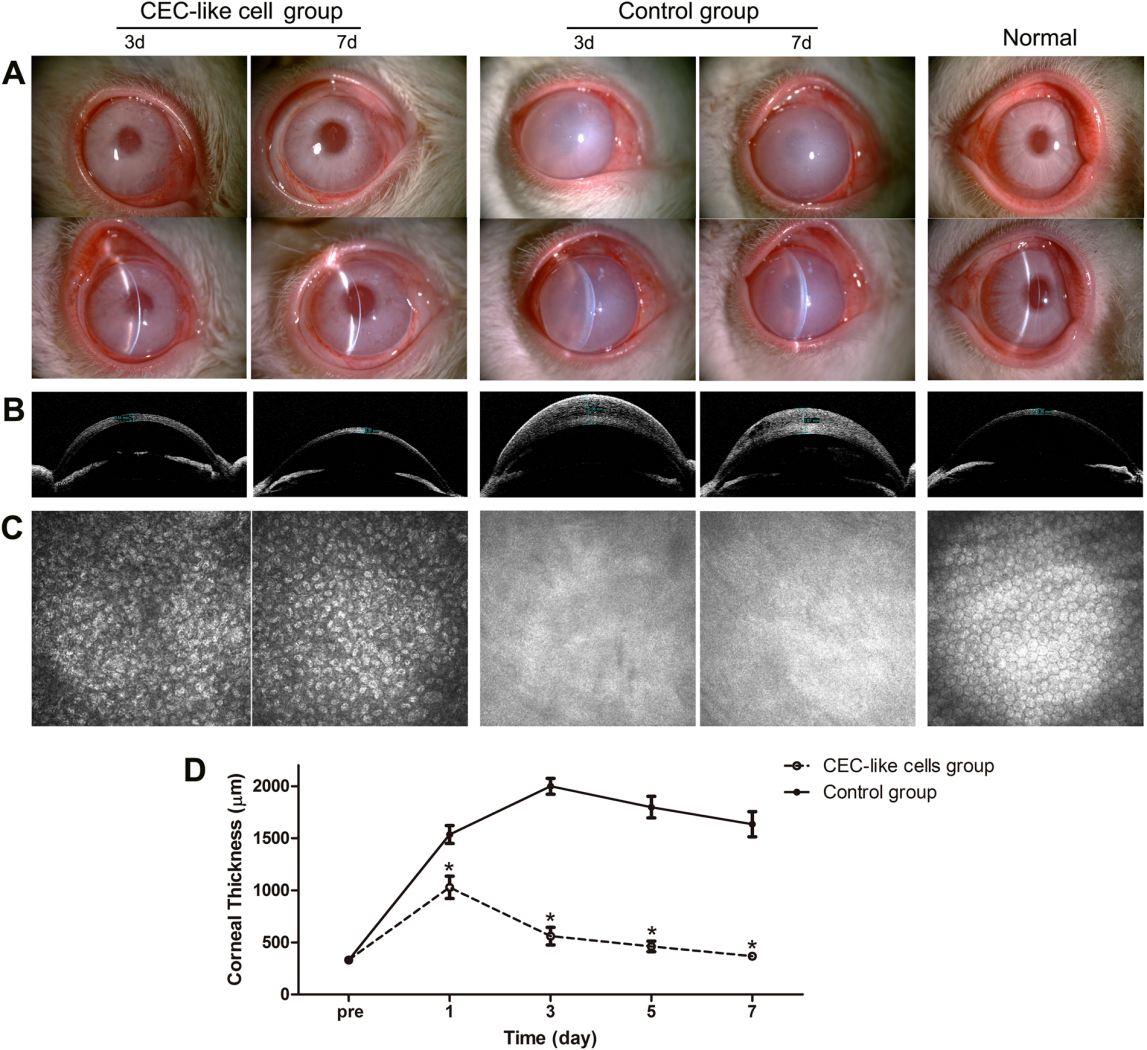
Clinical Observations of the CEC-like cell group and control group on day 3 and day 7 in rabbits. (**A**) Slit-lamp photographs showed that the clarity of the CEC-like cell group corneas significantly improved after injection, while corneal opacity and stromal edema were still serious in the control group. (**B**) Visante OCT showed significant corneal thickness differences in the CEC-like cell group and the control group. (**C**) Confocal microscope images confirmed full coverage of polygonal cells on the Descemet’s membrane in the CEC-like cell group. (**D**) Changes of the corneal thickness during clinical observation. There were significant differences in the corneal thickness between the control group (●) and the CEC-like cell group (○) on days 1, 3, 5, and 7. Results are mean ± S.D. (n = 10 per group) *p < 0.05 compared to control group.

The rabbit corneal endothelium was mechanically scraped from the Descemet’s membrane, and then CEC-like cells were injected into the anterior chamber. Slit-lamp photographs showed that the clarity of the CEC-like cell group corneas significantly improved after injection, and the pupil and iris texture could be seen. After only about 7 days, the corneas became clearly transparent, while corneal opacity and stromal edema were still serious in the control group (Fig. 4A). Visante OCT also showed that the corneal thickness rapidly decreased after injection of CEC-like cells (Fig. 4B). Confocal microscope images confirmed full coverage of polygonal cells on the Descemet’s membrane in the CEC-like cell group. However because of corneal opacity, this could not be clearly detected in the control group (Fig. 4C). It could be seen that the mean corneal thickness on 1, 3, 5, and 7 days (mean, 1.03 mm, 0.56 mm, 0.46 mm and 0.37 mm, respectively) in the CEC-like cell group were significantly less than that in the control group (P < 0.05). The corneal thickness on day 7 in the CEC-like cell group was almost as same as that before surgery (mean, 0.33mm) (Fig. 4D). Only mild inflammatory keratic or anterior chamber precipitates appeared after surgery and gradually disappeared a few weeks later.

### Histological Examination

**Figure 5:**
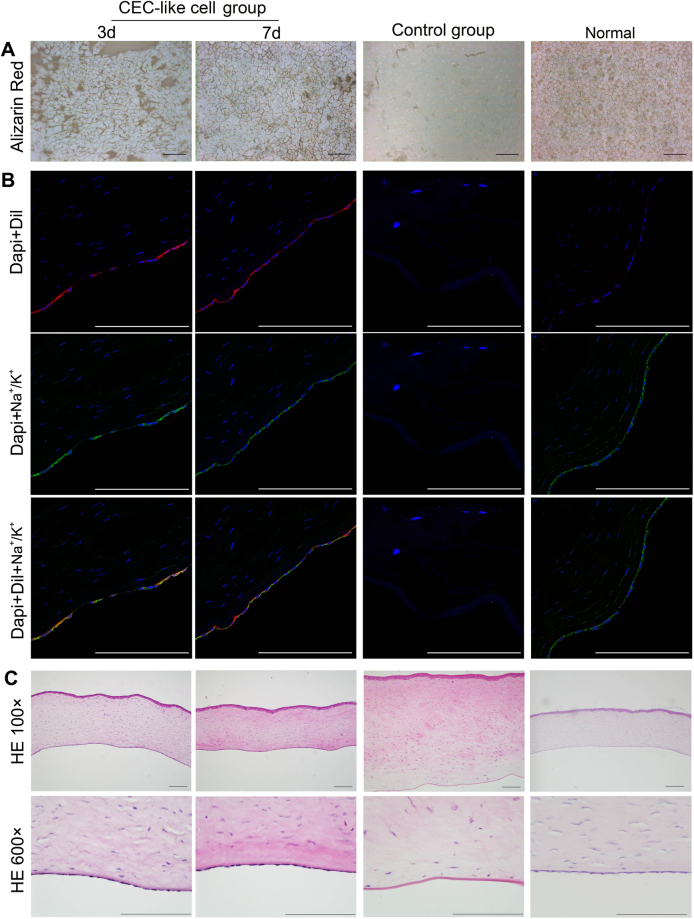
Histological examination of the CEC-like cell group and control group on 3 day and 7 day in rabbits. (**A**) Alizarin red and trypan blue staining. (**B**) Immunofluorescent staining of frozen section (blue: DAPI, red: Dil, green: Na^+^/K^+^ ATPase). (**C**) HE staining (top row:100×; bottown row: 600×). Scale bars: 100 μm.

Rabbits were killed on day 3 and day 7 to evaluate the CEC-like cells. Alizarin red and trypan blue staining showed that the monolayer polygonal CEC-like cells were alive and closely connected to each other (Fig. 5A). Fluorescent microscope examination of confirmed the existence of Dil-labeled CEC-like cells. These CEC-like cells almost covered the whole Descemet’s membranes. The immunofluorescent stain showed the Na^+^/K^+^ ATPase expression, indicating the pump function of CEC-like cells (Fig. 5B). Through HE staining it could be seen that the thickness of the cornea in the CEC-like cell group was similar to the normal group, while the cornea was very thick and edematous in the control group. Under magnified observation, the CEC-like cells tightly adhered to the posterior surface of the cornea in a monolayer. The Descemet’s membranes in the control group were bare and almost no cells were detected on day 7 (Fig. 5C). All the examination results in the CEC-like cell group were similar to results of the normal group, suggesting that the CEC-like cells play a role as normal CECs. Results of the histological examination were consistent with the clinical observation.

### Injection of CEC-like cells Enables Regeneration of the Cornea in the Primate Model

**Figure 6:**
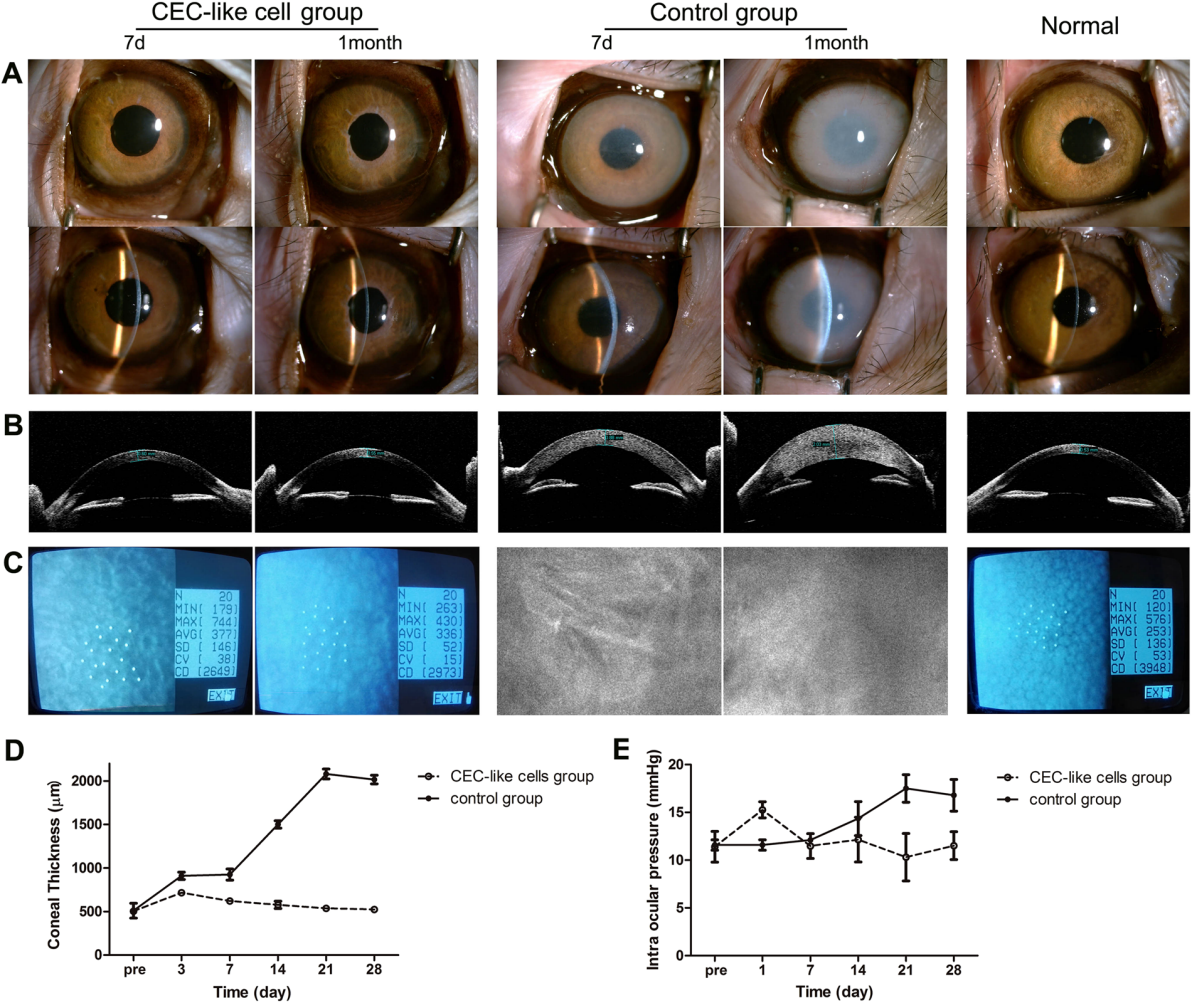
Clinical Observations of the CEC-like cell group and control group on day 7 and 1 month in monkeys. (**A**) Slit-lamp photographs showed that the corneas became significantly clearly transparent in the CEC-like cell group after injection, whereas corneal opacity and stromal edema became more and more serious in the control group. (**B**) Visante OCT showed significant corneal thickness differences in the CEC-like cell group and the control group. (**C**) Non-contact specular microscopy showed that CEC-like cells were in the form of a polygonal monolayer with a cell density of about 2500–3000 cell/mm^2^ in the CEC-like cell group. (**D**) Changes of the corneal thickness during clinical observation. (**E**) Changes of intraocular pressures during clinical observation. Results are mean ± S.D. (n_1_ = 3, n_2_ = 2).

We verified the transplantation experiment in monkeys. About 7 days after injection of CEC-like cells into monkey corneal dysfunction models, the corneas became significantly clearly transparent that the pupil and iris texture could be seen, while the corneas of the control group were cloudy and thick. One month later, the corneas of CEC-like cell group became clearer and thinner, whereas corneal opacity and stromal edema became more and more serious in the control group (Fig. 6A,B). Non-contact specular microscopy showed that CEC-like cells were in the form of a polygonal monolayer with a cell density about 2500–3000 cell/mm^2^ in CEC-like cell group. However because of the corneal opacity, this could not be clearly detected in the control group with a confocal microscope (Fig. 6C). It could be seen that the cornea became thinner from 3 to 28 days (mean, 0.72 mm, 0.62 mm, 0.58 mm, 0.54 mm, 0.52 mm) in the CEC-like cell group. On the contrary, corneal thicknesses in the control group were significantly thicker (mean, 0.91 mm, 0.93 mm, 1.50 mm, 2.08 mm, and 2.02 mm). It could also be seen that the corneal thickness from 21 to 28 days in the CEC-like cell group was almost the same as that before surgery (mean, 0.50 mm) (Fig. 6D). Similar to the observation in rabbits, only a mild inflammatory reaction existed and after application of steroid drugs it gradually disappeared. Intraocular pressures were found to be in the normal range in all groups (Fig. 6E).

### Long-term follow up

**Figure 7:**
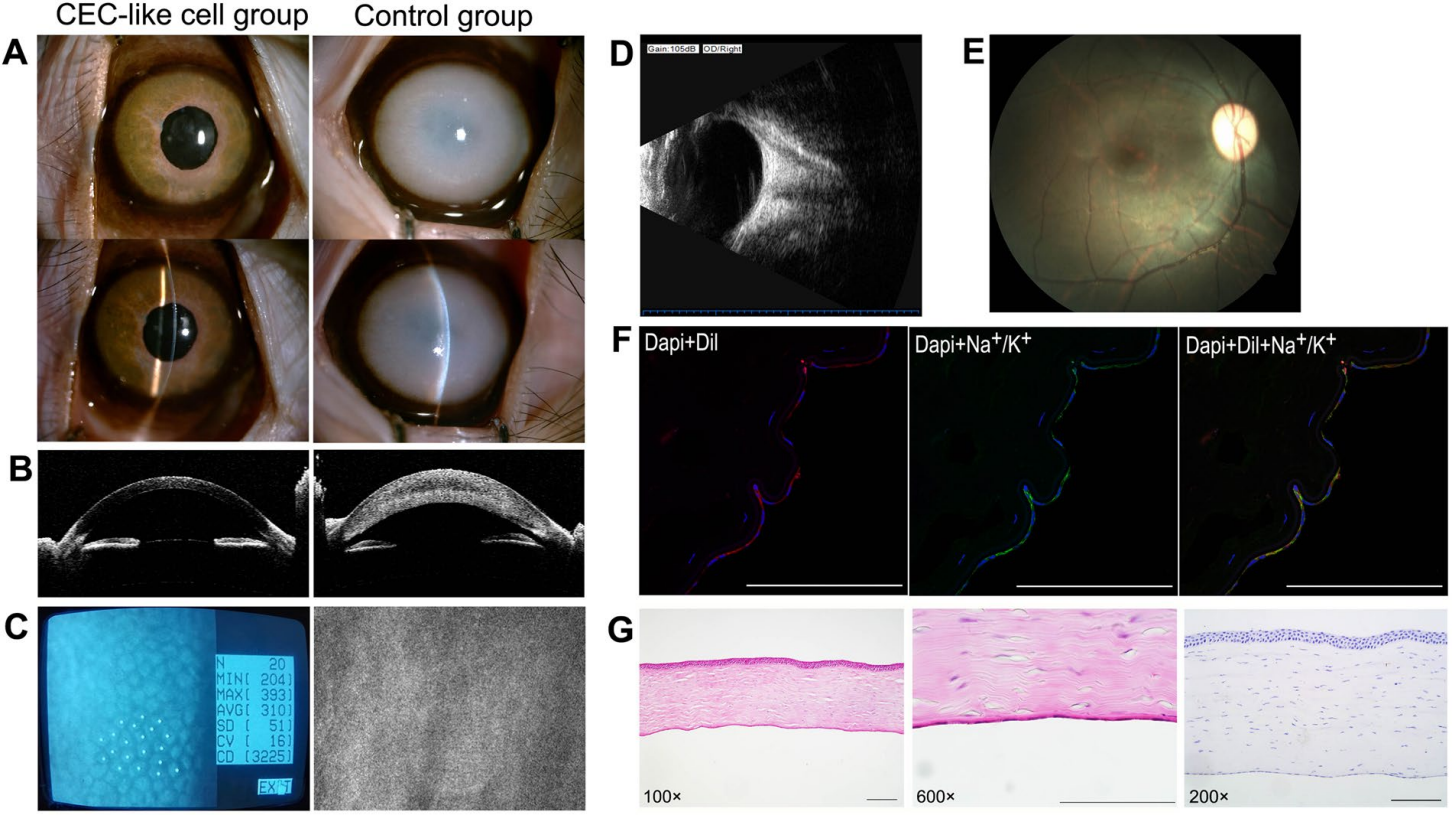
Three months after surgery in the monkeys. (**A**, **B**) The corneas of the CEC-like cell group were still transparent while the corneal opacity and stromal edema were still serious in the control group. (**C**) Non-contact specular microscopy. (**D**) B-mode ultrasound. (**E**) Fundus photography. (**F**) Immunofluorescent staining of frozen section (blue: DAPI, red: Dil, green: Na^+^/K^+^ ATPase). (**G**) HE staining and Immunohistochemical staining. Scale bars: 100 μm.

Three months after surgery in the monkeys, the cornea of the CEC-like cell group were still transparent while the corneal opacity and stromal edema were still serious in the control group. There were no new precipitates or vessels appeared during observation. (Fig. 7A,B). Polygonal CEC-like cells closely connected to each other in the form of a monolayer mosaic structure with a cell density about 3200 cell/mm^2^ (Fig. 7C). B-mode ultrasound and fundus photography showed no pathological changes of the surgical eyes (Fig. 7D,E). Dil-labeled CEC-like cells covered the Descemet’s membrane. Na^+^/K^+^ ATPase were also expressed in CEC-like cells, indicating the persistent pump function (Fig. 7F). HE staining showed that the cornea was almost normal and the CEC-like cells tightly connected to each other on the Descemet’s membrane and there were little inflammatory cells in the cornea. Immunohistochemical staining showed there were no apparent CD4^+^ cells in the cornea (Fig. 7G). In addition, no obvious tissue abnormalities were found in other parts of postoperative monkey (Supplementary Figure 1).

## Discussion

Previous studies demonstrated that CECs-like cells can be induced from embryonic stem cells^18^, adipose-derived stem cells^19^, bone marrow-derived endothelial progenitor cells^20^, neural crest cells^21^, or corneal stroma stem cells^22^ to treat corneal endothelial dysfunction. But these therapies have several problems, such as immune rejection, ethical issues, limited tissue accessibility, or lack of animal experiments^23^. SKPs may be the ideal seed cells for the derivation of CECs because of following reasons. First, CECs mainly originate from neural crest cells which have multiple differentiation ability *in vitro* ^11–13^. Coincidentally, SKPs were demonstrated to be embryonic neural-crest-related precursors that arise in the dermis during embryogenesis and persist into adulthood, and share many properties with neural crest stem cells^10^. SKPs are multipotent in that they can generate several functional cell types, such as neuronal cells, smooth muscle cells, adipocytes, chondrocytes, and osteocytes^8^. Second, SKPs can be isolated and expanded in large quantities from many parts of the skin such as the abdomen, limbs, foreskin, face, and scalp, and they can be subcultured for several passages and maintained for a long time when frozen. Third, SKPs are able to suppress the allogeneic activation of T-lymphocytes resulting in an improved health status of animals suffering from a graft-versus-host reaction^9^. These factors make SKPs an optimal cell source, so we selected them for CECs’ derivation.

Several previous articles demonstrated that conditioned medium or co-culturing with primary CECs could differentiate stem cells into CEC-like cells^18,20,21^, since cytokines secreted by cells play a main role. However these methods require fresh human corneas. As a novel method, we connected B4G12 cells with SKPs in a Transwell co-culture system to differentiate SKPs to CEC-like cells. Known as the immortalized cell population of human corneal endothelial cells, B4G12 cells are polygonal, strongly adherent forming a strict monolayer, and are characterized by junctional proteins, collagens, and Na^+^/K^+^ ATPase^24,25^. B4G12 cells have been widely used in different research aspects, such as evaluating sub-stratums supporting growth of CECs^26,27^ or studying the mechanism of factors promoting CECs proliferation^28^. But there are difficulties in application in clinics due to consideration of the long-term safety of B4G12 cells’ immortalized nature. We seeded SKPs in the lower chamber of 6-well tissue culture plate and seeded B4G12 in the upper Transwell insert. This avoided a direct connection between the two cell types but ensured movement of small molecules though the microporous membrane. We changed the Transwell insert every 4 days to avoid confluent B4G12 cells blocking the microporous membrane. In our preliminary experiments we found that laminin and chondroitin sulphate pre-coating was necessary for successful CEC-like cell differentiation. Moreover, using Transwell inserts was better than conditioned medium. The conditioned medium method could also produce CEC-like cells but the derivation period was about 2 weeks which is longer than co-culture method. More importantly, the morphology and markers’ expression were worse than CEC-like cells produced by the method of co-culture system (Supplementary Figure 2). B4G12 could secrete cytokines continuously in co-culture system, which is of vital importance in the derivation of CEC-like cells.

Since there were no exact phenotypic markers for CECs, we combined a lot of standards to identify CEC-like cells. First is the polygonal cell shape. We could see that the shape of CEC-like cells were similar to B4G12 cells in *in vitro* culture. Second, we selected several CEC markers, such as Na^+^/K^+^ ATPase and carbonic anhydrase type 2 (Car2) which function as a pump transporting excess fluid from corneal stroma, N-cadherin and intercellular tight junction (ZO-1) which function as a barrier, and collagen type VIII (Col8a2) and collagen type IV (Col4a2) which are components of the Descemet’s membrane^29,30^. Increased expression of these markers over time suggested the transformation from SKPs to CEC-like cells. In addition, many transcription factors and inductive signals coordinate to mediate neural crest cell migration in embryonic development. Pitx2 and FoxC1 are two of the transcription factors acting in the process, and their activation plays important roles in eye anterior segment development^22,31^. During our cell induction, Pitx2 and FoxC1 expression were obviously stimulated, which suggested that we successfully mimicked the developmental process from the neural crest to corneal endothelium *in vitro*. It was found that subcultured CEC-like cells maintained the monolayer polygonal cell shape and expression of major markers, and when culturing was continued for one month the status did not change. Using the serum-free medium is sufficient to maintain the condition. However, the mechanisms of our cell induction are not clear, so we should focus on this aspect in the future. To sum up, co-culturing SKPs and B4G12 with a Transwell system can differentiate SKPs into functional CEC-like cells and they are abundant when subcultured. Thus, this is a significant, simple and novel method that resolves the deficiency of human CECs.

Corneal endothelium transplantation experiments were carried out in rabbit and monkey corneal endothelial dysfunction models to evaluate the function of CEC-like cells *in vivo*. The proliferation ability of rabbit CECs may hinder the result. However, previous literature reported that rabbit CECs start to migrate from the far periphery to center for recovery 14 days after endothelial injury, while corneal opacity does not revert at day 14^32^. In this study, we scraped almost all endothelial cells on the Descemet’s membrane close to the corneal limbus to avoid the migration and proliferation of CECs. The cornea transparency was restored within 7 days before the migration of host endothelial cells, while the corneas of the control group were still opaque and edematous. Pathological examination also revealed that Dil-labeled CEC-like cells covered nearly the entire cornea within 3 days, suggesting the rapid proliferation of these cells. All these evidences proved that the restored corneal thickness and transparency in rabbits were due to the endothelial function of transplanted CEC-like cells. In addition, we have also tried *in vitro* and in *vivo* experiments using SKP from aged donors. The result was similar with that of young donors (Supplementary Figure 3). This showed the generality of our experiment.

The monkey is the species most similar to human^33^ and the endothelial defect in the monkey cornea is mainly covered by migration of the adjacent cells as in humans^34^. Similarities between the pattern of endothelial repair and endothelial decompensation in humans suggest that the monkey may be a better model than a rabbit^35^. Thus, we further verified animal experiments in monkey corneal endothelial dysfunction models. As can be seen, after injecting Dil-labeled CEC-like cells into the anterior chamber of the endothelial dysfunction model, the cornea became almost completely clear and the corneal thickness was restored within 1 month, while the control group presented severe stromal edema. CEC-like cells can form a close connection on the Descement’s membrane. The cell density gradually increased probably because of the proliferation of CEC-like cells. After 3 months, the corneas in the monkey CEC-like cell group remained in good condition and the endothelial density was almost the same as normal. According to all the evidence, we can conclude that our CEC-like cells could restore cornea transparency nearly as well as normal CEC cells *in vivo*.

It is worth mentioning that there was no apparent immune rejection after CEC-like cell transplantation. As we can see, only mild inflammatory keratic or anterior chamber precipitates appeared after surgery and gradually disappeared a few weeks later. During long-term observation, the corneas of the CEC-like cell group still maintained transparent and thickness, and there are no new precipitates or vessels appeared^36^. The HE and immunohistochemical staining also showed that there were little inflammatory cells in the cornea^37^. There might be three reasons. First, the literature reports that SKPs are able to suppress the allogeneic activation of T-lymphocytes, resulting in an improved health status of animals suffering from a graft-versus-host reaction^9^. Second, the anterior chamber is characterized with anterior-chamber-associated immune deviation (ACAID), permitting the long-term acceptance of a graft^38,39^. Third, we used serum free medium to culture CEC-like cells, avoiding a lot of immunogenic factors. Therefore, SKP-derived CEC-like cells have weak immunogenicity with no need for using large amount of anti-rejection drugs. This is benefit for the potential of clinical application in the future.

In the last decade, Descemet’s stripping endothelial keratoplasty (DSEK) and Descemet’s membrane endothelial keratoplasty (DMEK) have been used to treat corneal endothelial dysfunction^14^. But the surgical procedure is complex and difficult. In recent years, research suggested that the corneal endothelial dysfunction may be treated by injecting CECs supplemented with Rho-associated kinase (ROCK) inhibitor Y-27632 into the anterior chamber, and this method does not cause adverse effects, such as rejection, secondary glaucoma, or aberrant ectopic cell transplantation^15,16^. This may be because Y-27632 significantly promotes cell adhesion and proliferation, and inhibits the apoptosis of corneal endothelial cells^40,41^. In our study, CEC-like cells supplemented with Y-27632 were injected into the anterior chamber of rabbit and monkey corneal endothelial dysfunction models. Our transplantation experiment was very successful. The CEC-like cells exerted excellent therapeutic effect that the corneal endothelial dysfunction models recovered in a very short time. This may be a clinically relevant cell-based therapy for treating corneal endothelial dysfunction in the future.

Previously, Inagaki *et al*.^17^ had acquired CEC-like cells from SKPs. However, there are some difference between our work and their work. First, we used a different derivation method to obtain CEC-like cells from SKPs. Second, the transplantation method was different. We injected CEC-like cells into anterior chamber and it is more simple and easier to operate. Third, we also proved that these CEC-like cells were long-term functional in monkey corneal endothelial dysfunction models, which is of vital importance. Since rabbit corneal endothelial cells proliferate and regenerate, long-term observation is not possible. After three months, the monkey corneas remained in good condition and the endothelial density was almost the same as normal. What’s more, there was no apparent immune rejection during observation. This suggested that CEC-like cells could survive for a long time. It also indicated the value and prospect in clinical application in the future.

In conclusion, we successfully differentiated SKPs into CEC-like cells in a simple and efficient method. The CEC-like cells had similar morphology and characteristics to normal human CECs. We transplanted CEC-like cells into rabbit and monkey corneal endothelial dysfunction models, and the edematous corneas became transparent. Our derivation method is new and the treatment strategy is simple. It may be clinically applied in cellular replacement therapy or regenerative medicine in the future.

## Method

### Animals

Twenty New Zealand white rabbits weighing 2.0–4.0 kg (Xilingjiao Experimental Animal Breeding Center, Jinan, Shandong Province, China) and five rhesus monkeys weighting 2.5–3.5 kg (Hongli Medical Animal Experimental Research Center, Jinan, Shandong Province, China) were used for animal transplantation. All animals were treated in accordance to the ARVO (Association for Research in Vision and Ophthalmology) Statement for the Use of Animals in Ophthalmic and Vision Research. All animal experiments were approved by the Medical Ethics Committee of Qilu Hospital of Shandong University, China (approval number 12028).

### Cell culture

Samples were collected following written informed consents, and the study was performed in adherence to the Declaration of Helsinki. All experiments were approved by the Ethical Committee of Qilu Hospital of Shandong University and were conducted following the institutional guidelines (approval number 12028). SKPs were isolated and cultured as previously described^42,43^. Human skin was taken from 22 patients (age: 28.41 ± 10.45 years, mean ± S.D.) that underwent a double-eyelid operation. Briefly, freshly collected human skin samples were incubated with 0.2 mg/mL Liberase DH solution (Roche) for 12–24 h at 4 °C. Then, the epidermis was removed, isolated dermal tissue was minced into 1–2 mm^2^ small pieces, and incubated at 37 °C for 1–3 h depending on the size of sample. Cells were dissociated by repeated mechanical grinding of the tissue with a 10 ml disposable plastic pipette. For cultivation, a cell density of 25,000 or more cells/mL was used. The growth medium for SKPs consisted of a DMEM/F12 Nutrient Mixture (3:1; Thermo Fisher) supplemented with 1% penicillin/streptomycin (Sigma), 50 μg/mL fungizone, 2% B27 supplement (Thermo Fisher), 40 ng/mL human recombinant bFGF, and 20 ng/mL epidermal growth factor (both from Peprotech). Cell cultures were incubated at 37 °C in a 5% CO_2_-humidified atmosphere. The growth medium was replenished every 4–5 days. SKPs spheres were passaged every 1–2 weeks using 0.2 mg/mL Liberase DH solution. SKPs between passages 2 and 4 were used for further experiments. Donor information is described in Supplementary Table 1.

The immortalized HCEC population B4G12 was previously established by SV40-transfection in 2000^44^. B4G12 cells (generously provided by Monika Valtink) were cultured according to the previous protocol^24,25^. Briefly, B4G12 cells were cultured in human endothelial-SFM (Thermo Fisher) supplemented with 10 ng/ml human recombinant bFGF in T25 culture flasks coated with 10 mg/mL chondroitin sulphate and 10 μg/mL laminin (both from Sigma). Cells were passaged using 0.05% trypsin-0.02% EDTA at 37 °C for 10 min.

### Derivation of CEC-like cells from human SKPs

The co-culture system was used to differentiate human SKPs into CEC-like cells. Human SKPs spheres were separated to single cells by 0.05% trypsin-0.02% EDTA, and then they were plated into 6-well plates coated with 10 mg/ml chondroitin sulphate and 10 μg/ml laminin at a density of 5 × 10^5^ cells/ml. The immortalized HCECs population B4G12 cells were cultured at a density of 1 × 10^5^ cells/ml in six-well transwell inserts with 0.4 μm diameter pores and a polyethylene terephthalate membrane (Corning). Subsequently, the Transwell inserts were added to the 6-well plates and co-cultured with the human SKPs in human endothelial-SFM supplemented with 10 ng/ml human recombinant bFGF for 8 days. The medium was changed every 2 days and Transwell inserts were changed every 4 days. Our observations confirmed that the B4G12 cells plated in the upper compartment were unable to migrate through the membrane pores into the lower compartment, whereas the secreted cytokines were free to move. Moreover, cell morphology could be clearly observed every day through the Transwell inserts. The cells were cultured at 37 °C in a 5% CO_2_ humidified atmosphere. Cells were passaged when they became confluent, and cultured in human endothelial-SFM supplemented with 10 ng/ml bFGF.

### Immunofluorescent staining

Cells were fixed with 4% paraformaldehyde for 10 min and incubated in phosphate-buffered saline with 0.5% Triton X-100 (PBST) containing 1% goat serum albumin for 1 h. Primary antibodies were rabbit anti-nestin (1:100, Abcam), rabbit anti-vimentin (1:200, Abcam), mouse anti-Na^+^/K^+^ ATPase (1:100,millipore), and rabbit anti-zonula occludens-1 (ZO-1, 1:100, Santa Cruz). Cells were incubated with the primary antibodies for 1 h at 37 °C or overnight at 4 °C. Secondary antibodies (1:100, obtained from Beijing Zhongshan) coupled to FITC or TRITC were then applied for detection, and cells were stained with 4’, 6-diamidino-2-phenylindole (DAPI) to visualize the nuclei. In negative controls, PBS was substituted for the primary antibodies. Fluorescence was observed by using a fluorescent microscope.

### Western blotting

Total protein of SKPs and CEC-like cells was extracted, using 1% radio-immunoprecipitation assay lysis buffer (Beyotime) and quantified with the bicinchoninic acid protein assay kit (Beyotime). The protein was loaded onto a 8% sodium dodecyl sulfate–polyacrylamide electrophoresis gel, electrophoresed, and then transferred to a polyvinylidenedifluoride membrane (Millipore). Membranes were blocked with 5% nonfat dried milk at room temperature for 2 h, and then washed three times with TBST, and incubated with the primary antibody against rabbit anti- Na^+^/K^+^ ATPase (1:1000, CST), rabbit anti-zonula occludens-1 (ZO-1, 1:500, Santa Cruz), and rabbit anti-GAPDH(1:1000, Beijing Zhongshan) overnight at 4 °C. After immunoblotting with secondary antibodies (1:5,000, Beyotime) at room temperature for 1 h, the protein was detected with an enhanced chemiluminescent reagent (Millipore).

### Real-time reverse transcription polymerase chain reaction

Total RNA was extracted from SKPs and CEC-like cells using Trizol Reagent (Invitrogen) according to the manufacturer’s protocol. 1 μg sample of total RNA was reverse-transcribed to cDNA (complimentary DNA) with the ReverTra Ace a kit (Toyobo). qRT-PCR analysis was performed using the SYBR Green enzyme mixture (Toyobo) and the Applied Roche 480 Real-Time PCR system according to the manufacturer’s protocol. Primers used in the PCR are described in Supplementary Table 2.

### Rabbit Corneal Endothelial Dysfunction Model

Rabbits were under general anesthesia of pentobarbital sodium. Rabbits were divided into 2 groups (n = 10 each group) and the right eye was selected for our experiment. The corneal endothelium was mechanically scraped with a lacrimal passage irrigator (Shandong Weigao) from the Descemet’s membrane as previously described^15,16,45^. In the preliminary experiments, we confirmed that the Descemet’s membrane was intact, and the mechanically scraped area had nearly no cells on the Descemet’s membrane.

### Injection of Cultivated CEC-like cells into the Rabbit Eyes

CEC-like cells were labeled by Dil reagent as previously described^19^. Dil labeled CEC-like cells at a density of 2.0 × 10^5^ cells were suspended in 100 μl human endothelial-SFM supplemented with 3.2 μg of Y-27632 (ROCK inhibitor, Selleck). 100 μl aqueous humor was first extracted from the anterior chamber of the rabbit corneal endothelial dysfunction models and then cells were injected into the anterior chamber. Peribulbar and subconjuctival injection of triamcinolone and dexamethasone were given. Tobramycin and Dexamethasone were also given 3–4 times a day. 10 rabbits were injected with the cells as CEC-like cell group and the other 10 rabbits were not injected as the control group. After the surgery, the 20 rabbits were kept in a face-down position for 6 hours under general anesthesia. The left eyes of the rabbit were observed as the normal group.

Each surgical eye was photographed with a slit-lamp microscope (Carl Zeiss) and the central corneal thickness was measured with a Visante OCT (Carl Zeiss) at certain times. Images of CEC-like cells were taken and analyzed by confocal microscopy (HRT-II, Heidelberg Engineering).

### Histological Examination of Rabbit Eyes

Rabbits were sacrificed by an intravenous overdose of pentobarbital sodium 3 and 7 days after the transplantation. Postoperative eyes were removed and part of the cornea was embedded in OCT compound and sectioned to into 5 μM slices. The frozen slices were viewed under a microscope to detect the Dil signal and were subjected to immunofluorescent staining as previously described. The antibody was rabbit anti-Na^+^/K^+^ ATPase (1:100, CST). Part of cornea was double stained with alizarin red and trypan blue to show cell survival and borders. Another part of the cornea was fixed in 4% formaldehyde and subjected to HE staining.

### Injection of CEC-like cells into the Monkey Corneal Endothelial Dysfunction Model

Monkeys were under general anesthesia of ketamine hydrochloride. Monkey corneal endothelial pathological dysfunction models were created the same as the rabbit models. 50 μl aqueous humor was first extracted from the anterior chamber. Next, a 4.0 × 10^5^ density of Dil-labeled CEC-like cells suspended in 50 μl human endothelial-SFM supplemented with 1.6 μg of Y-27632 were injected into the anterior chamber of three monkeys as the CEC-like cell group. The other two monkeys were not injected as the control group. Peribulbar and subconjuctival injection of triamcinolone and dexamethasone were given. Tobramycin and Dexamethasone were also given 3–4 times a day. The eyes of all monkeys were kept in a face-down position for 6 hours under general anesthesia. The corneas were examined by a slit-lamp microscope, tenonometer, Visante OCT, confocal microscope, and non-contact specular microscopy at certain times after surgery. One monkey from the CEC-like cell group was euthanized at 3months after the injection. Postoperative eye were removed. Cornea was embedded and sectioned into 5 μM slices. The Dil signal, HE staining and immunofluorescent staining were carried out as described above. Immunohistochemical staining was performed using the anti-CD4^+^ antibody (cluster of differentiation 4, 1:100, Novus) following standard protocol.

### Statistical analyses

All data are presented as mean ± S.E.M or mean ± S.D. The Student’s t test was used to examine differences between the two groups. Comparisons among three or more groups were made using one-way ANOVA and post hoc analysis with the Bonferroni test. P < 0.05 was considered to be statistically significant. All data analyses were calculated by GraphPad Prism version 6 (GraphPad Software, Inc.).

## Electronic supplementary material

Supplementary information
