# Supplementary material for: Therapy of corneal endothelial dysfunction with corneal endothelial cell-like cells derived from skin-derived precursors
Source: Sci Rep. 2017 Oct 17;7:13400. doi: 10.1038/s41598-017-13787-1 (PMC5645363; doi:10.1038/s41598-017-13787-1)
Supplement: Supplementary file 1 — Supplementary information [file 41598_2017_13787_MOESM1_ESM.pdf]

Supplementary Information:

Therapy of corneal endothelial dysfunction with corneal endothelial cell-like cells derived from skin-derived precursors

Lin Shen [1, 2], Peng Sun [1], Canwei Zhang [1], Le Yang [3], Liqun Du [1], Xinyi Wu [1, *]

[1] Department of Ophthalmology, Qilu Hospital of Shandong University, Jinan, Shandong, 250012, PR China

[2] The Key Laboratory of Cardiovascular Remodeling and Function Research, Chinese Ministry of Education and Chinese Ministry of Health, Qilu Hospital of Shandong University, Jinan, Shandong, 250012, PR China

[3] Department of Vascular Surgery, Shandong Provincial Hospital Affiliated to Shandong University, Jinan, Shandong, 250021, PR China

* Corresponding author. Department of Ophthalmology, Qilu Hospital of Shandong University, Jinan, Shandong, 250012 PR China. E-mail: xywu8868@163.com.

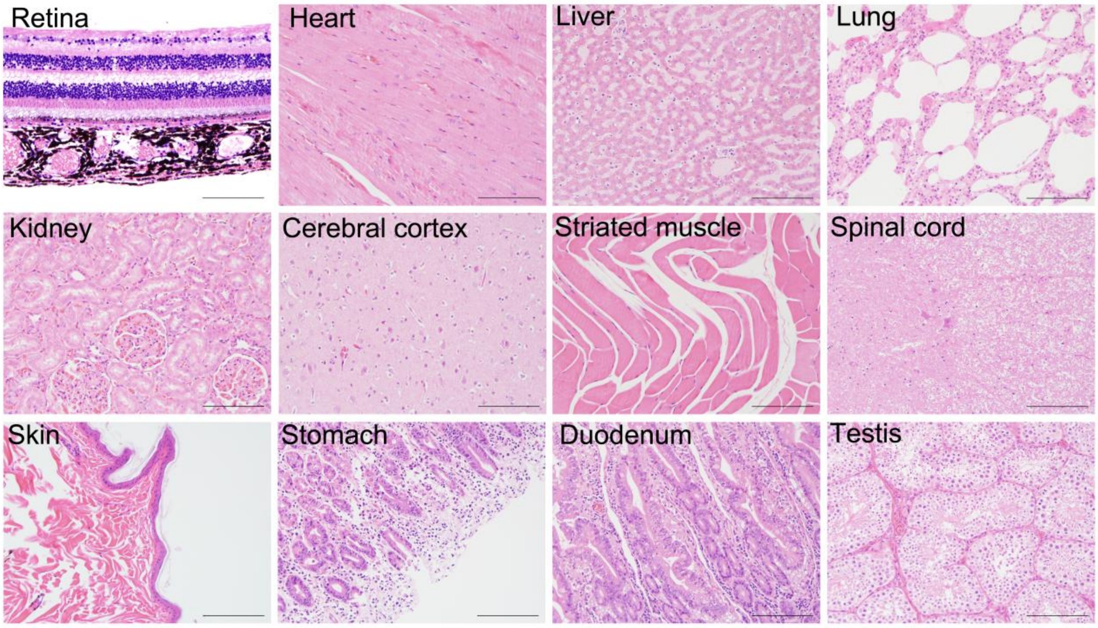

Supplemental Figure 1. HE staining showed that no obvious tissue abnormalities were found in other parts of postoperative monkey.

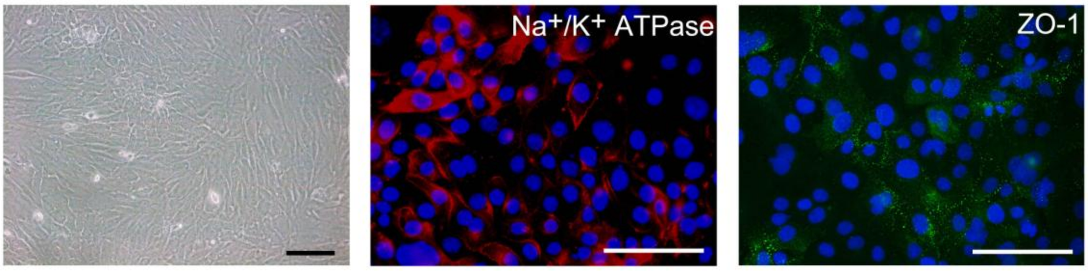

Supplementary Figure 2. The morphology and immunofluorescent staining of CEC-like cells derived from conditioned medium of B4G12 cells.

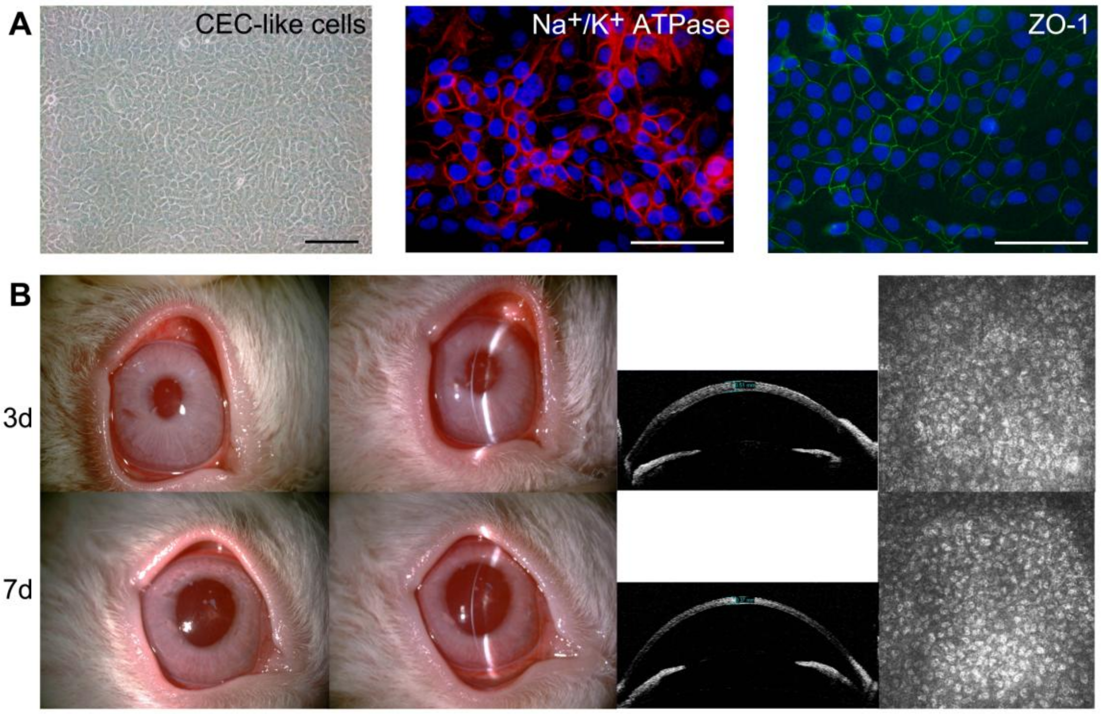

Supplementary Figure 3. The results of *in vitro* and *in vivo* experiments from older donors. (A) CEC-like cells were polygonal and formed a mosaic monolayer. Immunofluorescent staining showed the expression of Na^{+}/K^{+} ATPase and ZO-1 in CEC-like cells. (B) Slit-lamp photographs showed that the cornea became clear after CEC-like cells injection. Visante OCT showed a significant decrease of corneal thickness. Confocal microscope images showed the coverage of polygonal CEC-like cells.

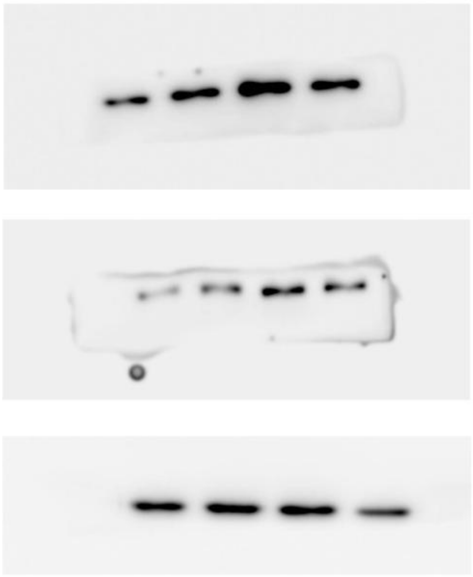

ZO-1

Na^{+}/K^{+} ATPase

GAPDH

Supplementary Figure 4. Full-length blots of Fig. 2E

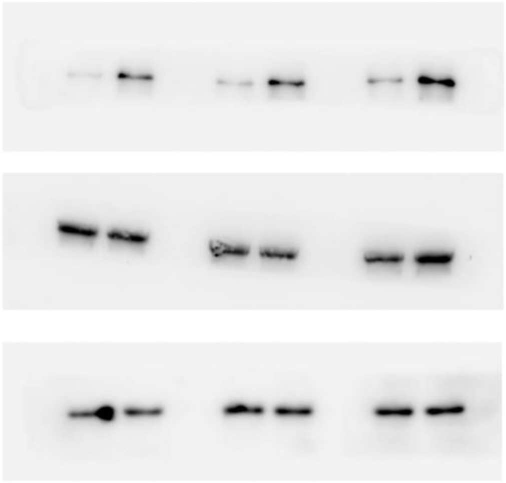

Supplementary Figure 5. Full-length blots of Fig. 3C

Supplemental Table 1: Donor information.

Serial Number	Age	Sex	Experiments				
			A	B	C	D	E
1	22	F	●				
2	24	F	●	●			
3	29	F	●		●		
4	18	F	●	●			
5	26	M	●	●			
6	25	F	●			●	
7	24	F	●		●		
8	27	F	●			●	
9	30	F	●	●	●		
10	22	M	●		●	●	
11	27	F	●			●	
12	36	M	●	●	●		
13	33	F	●			●	
14	22	F	●			●	
15	21	F	●		●		
16	36	F	●	●			
17	20	M	●			●	●
18	23	F	●			●	
19	28	F	●			●	●
20	19	F	●			●	●
21	52	M	●			●	
22	61	F	●			●	

A: Cell morphological assessment/Immunofluorescent staining
B: Real-time reverse transcription polymerase chain reaction
C: Western blotting
D: Rabbit experiment
E: Monkey experiment

Supplementary Table 2: Primers used for qRT-PCR analysis.

	**Forward**	**Reverse**
Na^+/K^+ ATPase	5'-ACAGACTTGAGCCGGGGATTA-3'	5'-TCCATTCAGGAGTAGTGGGAG-3'
ZO-1	5'-ACCAGTAAGTCGTCCTGATCC-3	5'-TCGGCCAAATCTTCTCACTCC-3'
N-cadherin	5'-AGCCAACCTTAACTGAGGAGT -3'	5'-GGCAAGTTGATTGGAGGGATG -3'
CA2	5'-GGGTACGGCAAACACAACG -3'	5'-GGCTGTATGAGTGTCGATGTC-3'
Col4a2	5'- TTATGCACTGCCTAAAGAGGAGC -3'	5'- CCCTTAACTCCGTAGAAACCAAG -3'
Col8a2	5'-TGCTACTGAAATGCCTCTACCG -3'	5'-GAGTCCTGGCTTTCCCATGC-3'
FoxC1	5'-TGTTCGAGTCACAGAGGATCG -3'	5'-ACAGTCGTAGACGAAAGCTCC-3'
Pitx2	5'-GCCAAGGGCCTTACATCCG -3'	5'-GGTGGGGAAAACATGCTCTG-3'
GAPDH	5'-TGATGACATCAAGAAGGTGGTGAAG-3'	5'-TCCTTGGAGGCCCATGTGGGCCAT-3'
